# Draft Genome Sequence of Cyclobacterium marinum Strain Atlantic-IS, Isolated from the Atlantic Slope off the Coast of Virginia, USA

**DOI:** 10.1128/MRA.01089-19

**Published:** 2019-12-12

**Authors:** Indu Sharma, Michael D. Lee

**Affiliations:** a Biological Sciences, Hampton University, Hampton, Virginia, USA; b Exobiology Branch, NASA Ames Research Center, Mountain View, California, USA; c Blue Marble Space Institute of Science, Seattle, Washington, USA; University of Southern California

## Abstract

Here, we report a draft genome sequence for Cyclobacterium marinum strain Atlantic-IS, isolated from the Atlantic slope off the coast of Virginia. The whole-genome sequence will help us understand its adaptive metabolic responses to diverse C sources in low-nutrient environments.

## ANNOUNCEMENT

**FIG 1:**
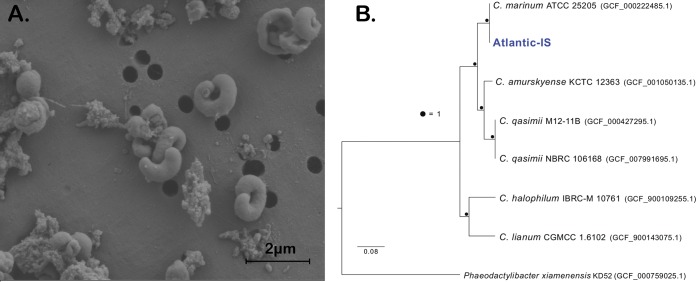
Cyclobacterium marinum Atlantic-IS. Scanning electron micrograph showing circular-like morphology (A) and estimated maximum likelihood phylogenomic tree based on concatenated amino acid alignments of 90 single-copy core genes specific for *Bacteroidetes* (B).

Belonging to the phylum *Bacteroidetes* and the class *Flavobacteriia*, the genus Cyclobacterium gets its name from its members’ circular-like morphology. *Cyclobacterium* spp. are Gram-negative, nonmotile heterotrophs that have been isolated from various marine and sediment environments (1–5). Here, we introduce the draft genome sequence of Cyclobacterium marinum strain Atlantic-IS. This strain was isolated from bottom water collected near the ocean floor at a depth of 258 m, global positioning system (GPS) coordinates 37°05′66.94″N, 73°51′13.06″W. The sampling site is located off the coast of Virginia along the Atlantic slope near Washington Canyon. Recently, a U.S. Geological Survey (USGS) survey mapped several patchy cold seeps in the surrounding areas (6). The water sample was collected from 0.75 m above the ocean floor using a Rosette sampler. The conductivity-temperature-depth (CTD) data (Sea-Bird Scientific) were 35.2 salinity, 10.6°C water temperature, and 4.1 mg/liter dissolved oxygen. A 100-μl seawater sample was plated on medium containing 1 part filtered natural seawater from a 258-m depth mixed with 1 part artificial seawater complete medium (342.2 mM NaCl, 14.8 mM MgCl_2_, 1 mM CaCl_2_, 6.71 mM KCl, 5 g Bacto-tryptone, 1 g peptone, 5 mM MOPS [pH 8.0], 0.03% glycerol) and agar at a final concentration of 1.5%. The plate was incubated at room temperature in the dark. On day 7, a small red-pigmented colony was selected, and an axenic culture was obtained for downstream processing. Amplification of the 16S rRNA gene using universal primers 8F and 1510R and sequencing confirmed that the isolate is a member of the *Cyclobacterium* genus (7, 8). Cell morphology was observed using a Zeiss Supra40VP scanning electron microscope (SEM) (Fig. 1A) (9).

For whole-genome shotgun sequencing, genomic DNA was extracted using the UltraClean microbial DNA isolation kit (Mo Bio Laboratories, Inc.), and library preparation was performed using Illumina’s Nextera XT library prep kit. Illumina adapters and unique 8-bp dual indexes were ligated to 1 ng of DNA with no protocol adaptations. The library quality was checked on an Agilent Bioanalyzer 2100 with a high-sensitivity DNA analysis kit. The library was normalized, multiplexed, pooled, and subsequently purified on a 2% agarose gel with GelStar stain (Lonza) to aid with visualization. DNA was size selected between 250 and 400 bp for extraction using a QIAquick gel extraction kit (Qiagen) for sequencing. The final purified library was then quality checked and quantified on a Bioanalyzer, followed by sequencing using 150-bp paired-end chemistry on a NextSeq 550 platform. A total of 13,034,355 read pairs were generated.

Trimmomatic v0.39 (10) was used to quality filter and trim the raw reads with the following parameters: LEADING:3 TRAILING:3 SLIDINGWINDOW:4:20 MINLEN:80. A total of 6,786,495 read pairs were retained, as well as a combined 4,881,460 individual forward and reverse reads, which were used to generate the genome assembly. For all the following programs, default settings were used. Assembly was performed using SPAdes v3.13.1 (11), and assembly summary statistics were generated with QUAST v5.0.2 (12). The draft genome total length is 6,156,343 bp, composed of 136 contigs (*N*_50_, 314,733 bp, *L*_50_, 8), with an average GC content of 38.46%. Recruiting the quality-filtered and trimmed reads back to the assembly with Bowtie2 v2.3.4 (13) yielded a mean genome coverage of ∼447-fold. Based on annotation using the NCBI Prokaryotic Genome Annotation Pipeline (PGAP) (14), the assembly contains 4,690 complete coding-sequence predictions, 37 tRNAs, and 75 pseudogenes. GToTree (v1.4.5) was used to create a phylogenomic tree of the currently available *Cyclobacterium* assemblies based on the concatenated alignment of 90 single-copy genes specific to the *Bacteroidetes* phylum (Fig. 1B) (15–19). The average nucleotide identity between Atlantic-IS and the *C. marinum* type strain (ATCC 25205) was 98.8%, with ∼90% of the two genomes aligning (fastANI v1.2) (20).

### Data availability.

This whole-genome shotgun project was deposited in NCBI’s GenBank under accession number VTDH00000000. Raw sequencing reads were deposited in NCBI’s Sequence Read Archive under accession number SRR10054430. The 16S rRNA gene amplicon sequence was deposited in GenBank under accession number MN164649.
